# Evidence From Men for Ovary-independent Effects of Genetic Risk Factors for Polycystic Ovary Syndrome

**DOI:** 10.1210/clinem/dgab838

**Published:** 2021-11-19

**Authors:** Jia Zhu, Natàlia Pujol-Gualdo, Laura B L Wittemans, Cecilia M Lindgren, Triin Laisk, Joel N Hirschhorn, Yee-Ming Chan

**Affiliations:** 1 Division of Endocrinology, Boston Children’s Hospital, Boston, MA 02115, USA; 2 Program in Medical and Population Genetics, The Broad Institute of MIT and Harvard, Cambridge, MA 02142, USA; 3 Department of Pediatrics, Harvard Medical School, Boston, MA 02115, USA; 4 Estonian Genome Centre, Institute of Genomics, University of Tartu 51010, Tartu, Estonia; 5 Department of Obstetrics and Gynecology, PEDEGO Research Unit, Medical Research Centre, Oulu University Hospital, University of Oulu FI-90014, Oulu, Finland; 6 Big Data Institute, Li Ka Shing Centre for Health Information and Discovery, University of Oxford, Oxford OX3 ZFZ, UK; 7 Nuffield Department of Women’s and Reproductive Health, University of Oxford, Oxford OX3 9DU, UK; 8 The Wellcome Trust Centre for Human Genetics, University of Oxford, Oxford OX3 7FZ, UK; 9 Department of Genetics, Harvard Medical School, Boston, MA 02115, USA

**Keywords:** polycystic ovary syndrome, PCOS, polygenic risk score, obesity

## Abstract

**Context:**

Polycystic ovary syndrome (PCOS) is characterized by ovulatory dysfunction and hyperandrogenism and can be associated with cardiometabolic dysfunction, but it remains unclear which of these features are inciting causes and which are secondary consequences.

**Objective:**

To determine whether ovarian function is necessary for genetic risk factors for PCOS to produce nonreproductive phenotypes.

**Design, Setting, and Participants:**

Cohort of 176 360 men in the UK Biobank and replication cohort of 37 348 men in the Estonian Biobank.

**Main Outcome Measures:**

We calculated individual PCOS polygenic risk scores (PRS), tested for association of these PRS with PCOS-related phenotypes using linear and logistic regression and performed mediation analysis.

**Results:**

For every 1 SD increase in the PCOS PRS, men had increased odds of obesity (odds ratio [OR]: 1.09; 95% CI, 1.08-1.10; *P *= 1 × 10^-49^), type 2 diabetes mellitus (T2DM) (OR: 1.08; 95% CI, 1.05-1.10; *P *= 3 × 10^-12^), coronary artery disease (CAD) (OR: 1.03; 95% CI, 1.01-1.04; *P *= 0.0029), and marked androgenic alopecia (OR: 1.03; 95% CI, 1.02-1.05; *P *= 3 × 10^-5^). Body mass index (BMI), hemoglobin A1c, triglycerides, and free androgen index increased as the PRS increased, whereas high-density lipoprotein cholesterol and SHBG decreased (all *P *< .0001). The association between the PRS and CAD appeared to be completely mediated by BMI, whereas the associations with T2DM and marked androgenic alopecia appeared to be partially mediated by BMI.

**Conclusions:**

Genetic risk factors for PCOS have phenotypic consequences in men, indicating that they can act independently of ovarian function. Thus, PCOS in women may not always be a primary disorder of the ovaries.

Polycystic ovary syndrome (PCOS) is a common polygenic disorder affecting 6% to 10% of women of reproductive age (1). Cardiometabolic dysfunction, as evidenced by insulin resistance, dyslipidemia, and obesity, is often a significant comorbidity with long-term effects on cardiometabolic health (1, 2). Despite extensive physiologic and genetic studies, the pathogenesis of PCOS remains incompletely understood.

PCOS is a heterogeneous disorder, which raises challenges in understanding its pathophysiology. Studies show that PCOS is associated with perturbation of presumed ovarian-related factors and nonovarian factors (3). However, it remains to be determined which of these are the inciting events and which are the secondary consequences.

Historically, PCOS has been conceptualized as a disorder of reproductive-aged women, with ovarian dysfunction as a key feature (1). However, many have since suggested that PCOS may not always be primarily a disorder of the female reproductive system (4, 5). Indeed, male first-degree relatives of women with PCOS have increased rates of cardiometabolic dysfunction (4-9). A Mendelian randomization analysis suggested that genetic variants associated with androgenic alopecia in men may play a causal role in PCOS (3). These observations in men suggest that genetic variants associated with PCOS may not act through alterations in ovarian function to influence the development of the cardiometabolic and androgenic features of PCOS.

The largest genome-wide association study (GWAS) meta-analysis of PCOS risk in 2018 identified numerous genetic loci that can be used to generate polygenic risk scores (PRSs) that quantify the susceptibility for PCOS because of common genetic variants (3, 10, 11). In this study, we optimized a PRS for PCOS and examined associations between this PRS and cardiometabolic and androgenic phenotypes in the absence of ovarian function (ie, in men).

## Methods

Standards for the development, validation, and association testing of a PRS for PCOS were followed as described here and in the Supplemental Materials (12, 13).

### Study Cohorts

The UK Biobank (UKBB) is a population-based cohort of ~500 000 individuals in the United Kingdom aged 40 to 69 years at recruitment (14). Additional characteristics are reported in the Supplemental Materials (12). At the time of analysis, there were 383 212 unrelated individuals (176 360 genetic males and 206 852 genetic females) of European ancestry with genotype data available.

The replication cohort consisted of men and women from the Estonian Biobank (EstBB), a population-based biobank with > 200 000 participants. Additional characteristics are reported in the Supplemental Materials (12). At the time of analysis, there were 91 384 unrelated individuals (37 348 genetic males and 54 036 genetic females) of European ancestry with genotype data available.

### PCOS PRS Calculation

To generate PRSs for PCOS, we used summary statistics from the largest published GWAS meta-analysis for PCOS, which included 113 238 women of European ancestry (3, 15, 16). We compared the following 3 methods for calculating PRSs, which are further described in the Supplemental Materials (12): (1) PRSice-2 software; (2) PRS-CS software; and (3) PRS-CS software with modification to incorporate probabilistic genotype dosages generated by imputation (10, 11).

### Optimizing the PRSs in Women in the UKBB and Validation in the EstBB

To optimize the performance of the PCOS PRSs, we identified women in the UKBB with a likely diagnosis of PCOS determined by self-report during a verbal interview with a trained nurse, primary-care clinical events, and/or International Classification of Diseases, 9th and 10th revisions, codes (Supplemental Materials; (12)). Because PCOS is a disorder of reproductive-age women and the UKBB has a relatively older age at recruitment (40-69 years), some women in the UKBB may have menstrual irregularity from menopause rather than PCOS. We therefore further narrowed our analysis to women ≤ 50 years old with no reported history of menopause on questionnaire.

The 3 methods to calculate PRSs were then applied to these cohorts. For each method, we calculated the phenotypic variance explained for PCOS using a logistic regression model with the first 10 principal genetic components to adjust for potential population stratification, array number, and assessment center as covariates. To assess the risk of PCOS with increasing PCOS genetic risk score, we separated participants by quintile of PRSs and used logistic regression to assess the odds ratio (OR) of PCOS for each quintile using the lowest quintile as reference. We used the best-performing method (highest *R*^2^) for all subsequent analyses.

We validated the optimized PCOS PRSs in women in the EstBB, who were not included in the GWAS meta-analysis for PCOS that was used to generate the PCOS PRSs in this study (3). Women with PCOS were identified by the International Classification of Diseases, 10th revision, code E28.2, and women who did not have a diagnosis of PCOS served as controls. We then compared the risk of PCOS with increasing PCOS PRSs between women in the UKBB and the EstBB with 2 heterogeneity tests, the I^2^ and Cochran’s Q test.

### Cohorts and Phenotypes in Men

We used the PRS method that was optimized in women to calculate PRSs for men in both the UKBB and EstBB. The resulting PRSs were scaled to a mean of 0 and an SD of 1.

Cardiometabolic and androgenic phenotypes were based on a composite of anthropometric measurements, self-reported measures, diagnosis and procedure codes from hospitalization records, medication use, and age at diagnosis (Supplemental Materials; (12; 17-20).

### Statistical Analysis

For continuous phenotypes, we used linear regression to analyze the association between the phenotypes and the PRSs. We also grouped participants by PRS decile and determined the average for each phenotype (with 95% CIs) within each decile. For dichotomous outcomes, we used logistic regression to calculate the OR of the outcome per 1 SD increase of the PRS. We also used logistic regression to compare the odds of the outcome for participants with high (top 20%, 10%, 5%, 1%), low (bottom 20%, 10%, 5%, 1%), and middle genetic risk scores (middle 20%) with the remainder of the cohort.

All analyses were adjusted for age, age squared, genotyping array, and the first 10 genetic principal components to adjust for potential population stratification. For participants in the UKBB, analyses were additionally adjusted for the UKBB assessment center to control for genetic relatedness and other confounding factors. For participants in the EstBB, all analyses were adjusted for batch effects. All analyses were performed with and without body mass index (BMI) as a covariate.

To further address the role of BMI, we conducted causal mediation analysis to assess the indirect effect of BMI in the association between the PRSs and the outcomes of interest (Supplemental Materials; (12)). All statistical analyses were conducted using R v3.5.0. The R package “mediation” was used to conduct causal mediation analysis (21).

## Results

### Optimization of a PCOS PRS in UKBB Females and Validation in EstBB Females

To calculate a PRS for PCOS, we started with the estimated effect sizes for common genetic variants determined in the largest GWAS meta-analysis for PCOS on females of European ancestry (3). To optimize the PRS, 3 different algorithms as well as ranges of tuning parameters (see Materials and Methods) were tested for association with the PCOS phenotype in a dataset of 206 852 unrelated females of European ancestry in the UKBB. In this cohort study, we note that the women identified as having PCOS (N = 1003 cases, prevalence of 0.5%) are almost certainly a subset of the women who have PCOS and may also include women with reproductive dysfunction because of menopause. Although optimization of a PRS does not require precise diagnostic distinctions, we also refined the set of women with a likely diagnosis of PCOS in the UKBB by analyzing a sub-cohort of 50 612 females who were ≤ 50 years of age with no history of menopause, resulting in an increased prevalence of PCOS (N = 669 cases, prevalence of 1.3%; Supplemental Materials, Fig. S1A; (12). The PRSs produced by the 3 algorithms tested were all normally distributed and showed significant association with PCOS in both the total cohort and the subcohort (all *P *< .01), with *R*^2^ ranging from 0.0014 to 0.0052 (Supplemental Materials, Table S1; (12)). The PRS-CS method modified to incorporate probabilistic genotype dosage performed the best in the total cohort (*R*^2^ = 0.0045) and the subcohort (*R*^2^ = 0.0052) and was selected for subsequent analyses (10, 11). Women who had a PRS in the top quintile had 71% greater odds of having PCOS compared with those in the bottom quintile (OR: 1.71; 95% CI, 1.33-2.19; *P* = 3 × 10^-5^; Supplemental Materials, Fig. S1C; (12)); thus, this PRS successfully captures genetic risk for PCOS in women in the UKBB.

We then validated the association between the optimized PCOS PRS with PCOS in 54 036 unrelated females of European ancestry in the EstBB (3651 cases of PCOS and 50 385 controls). Women who had a PRS in the top quintile had 45% greater odds of having PCOS compared with those in the bottom quintile (OR: 1.45; 95% CI, 1.29-1.63; *P *= 2 × 10^-10^; Supplemental Materials, Fig. S2; (12)). This OR was not significantly different from the estimates from UKBB (*I*^2^ = 28%, Cochran’s Q *P* value for heterogeneity = .24).

### Associations Between Polygenic Risk for PCOS and Cardiometabolic Phenotypes in Men

We determined the relationship between the PRS and cardiometabolic phenotypes in 176 360 unrelated men of European ancestry in the UKBB. Based on composite algorithms incorporating self-reported measures, diagnosis and procedure codes from hospitalization records, and medication use, we identified 44 499 cases of obesity, 15 857 of coronary artery disease (CAD), 11 730 of type 2 diabetes mellitus (T2DM), and 87 830 of marked androgenic alopecia (Table 1).

**Table 1.** Ascertainment of cardiometabolic and androgenic phenotypes in men in the UK Biobank (N = 176 360)

Cardiometabolic phenotypes	Mean ± SD or cases/controls	N
*Continuous*		
BMI (kg/m^2^)	27.8 ± 4.2	175 708
Waist-to-hip ratio	0.94 ± 0.07	176 000
HbA1c (mmol/mol)	36.3 ± 7.3	168 088
HDL cholesterol (mmol/L)	1.29 ± 0.31	155 355
Triglycerides (mmol/L)	1.98 ± 1.15	168 122
*Dichotomous* ^*a*^		
Obesity	44 499/131 861	176 360
Type 2 diabetes mellitus	11 730/97 146	108 876
Coronary artery disease	15 857/160 503	176 360
Androgenic phenotypes		
*Continuous*		
SHBG (nmol/L)	40 ± 17	154 245
Free androgen index^*a*^	33 ± 14	153 352
*Dichotomous*		
Marked androgenic alopecia^*a*^	31 852/55 978	87 830

Abbreviations: BMI, body mass index; HbA1c, hemoglobin A1c; HDL, high-density lipoprotein.

^*a*^See supplemental materials for definitions and ascertainment details.

**Figure 1.**
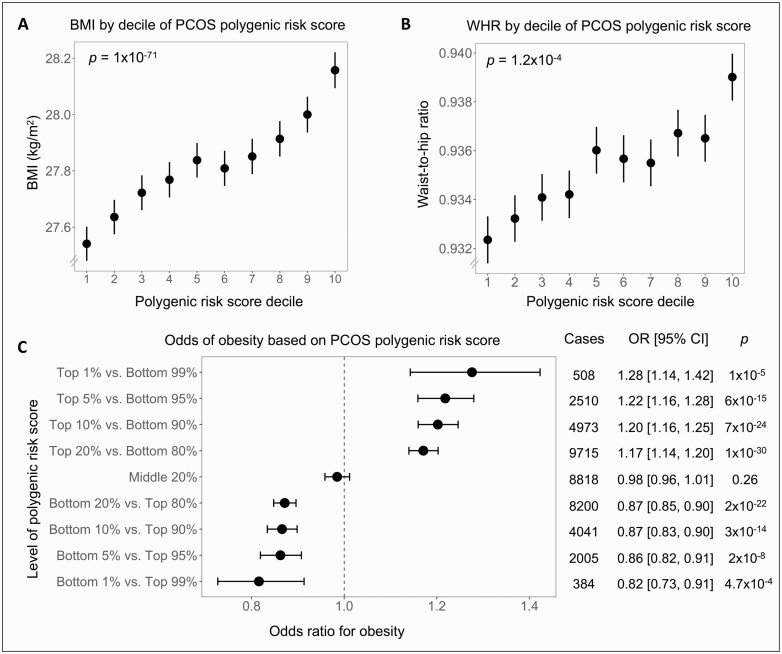
Association of polycystic ovary syndrome (PCOS) polygenic risk score with body mass index (BMI), waist-to-hip ratio (WHR), and obesity. (A-B) 176 360 unrelated men in the UK Biobank were separated into 10 deciles by polygenic risk score for PCOS. (A) BMI and (B) WHR increased across these deciles (*P* value for linear regression shown). (C) Odds ratios for obesity (defined by BMI ≥ 30 kg/m^2^) were calculated by comparing those with a high, middle, or low polygenic risk score with the remainder of the cohort using logistic regression. Error bars indicate 95% CI. All analyses were adjusted for age, age squared, genotyping array, and the first 10 genetic principal components.

For BMI, we observed a 0.20 kg/m^2^ increase for every 1 SD increase in the PRS (*P *= 1 × 10^-71^). To assess abdominal adiposity, we also examined the waist-to-hip ratio (WHR) controlled for BMI in addition to the standard covariates, and we observed a 4.8 × 10^-4^ increase in WHR per 1 SD of PRS (*P *= 1.2 × 10^-4^). We further grouped participants into deciles by their PRS and observed a gradient of increasing BMI and WHR across increasing deciles (Figs. 1A, 1B).

Next, we assessed obesity (defined as BMI ≥ 30 kg/m^2^) and observed a 9% increase in odds of obesity per 1 SD of PRS (OR: 1.09; 95% CI, 1.08-1.10; *P *= 1 × 10^-49^). We also observed a higher risk of obesity with higher PRS across a range of percentile cutoffs (Fig. 1C).

**Figure 2.**
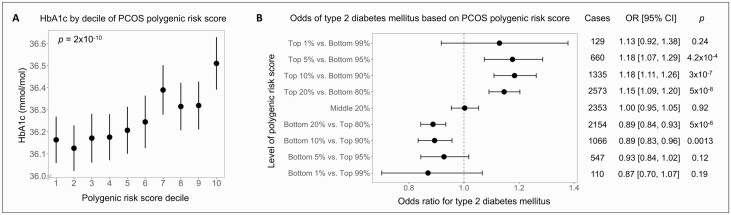
Association of polycystic ovary syndrome (PCOS) polygenic risk score and hemoglobin A1c (HbA1c) and type 2 diabetes mellitus. (A) HbA1c increased across deciles of the polygenic risk score for PCOS (*P* value for linear regression shown). (B) Odds ratios for type 2 diabetes mellitus were calculated by comparing those with a high, middle, or low polygenic risk score with the rest of the cohort using logistic regression. Error bars indicate 95% CI. All analyses were adjusted for age, age squared, genotyping array, and the first 10 genetic principal components.

Men who carried a high PRS also had higher hemoglobin A1c (HbA1c; 0.12 mmol/mol or 0.011% increase per 1 SD of PRS, *P *= 2 × 10^-10^), and HbA1c increased across increasing deciles of the PRS (Fig. 2A). We also assessed the association with T2DM and found an 8% increase in odds of T2DM per 1 SD of PRS (OR: 1.08; 95% CI, 1.05-1.10; *P *= 3 × 10^-12^) and an increased odds of T2DM with higher PRS across a range of percentile cutoffs (Fig. 2B).

**Figure 3.**
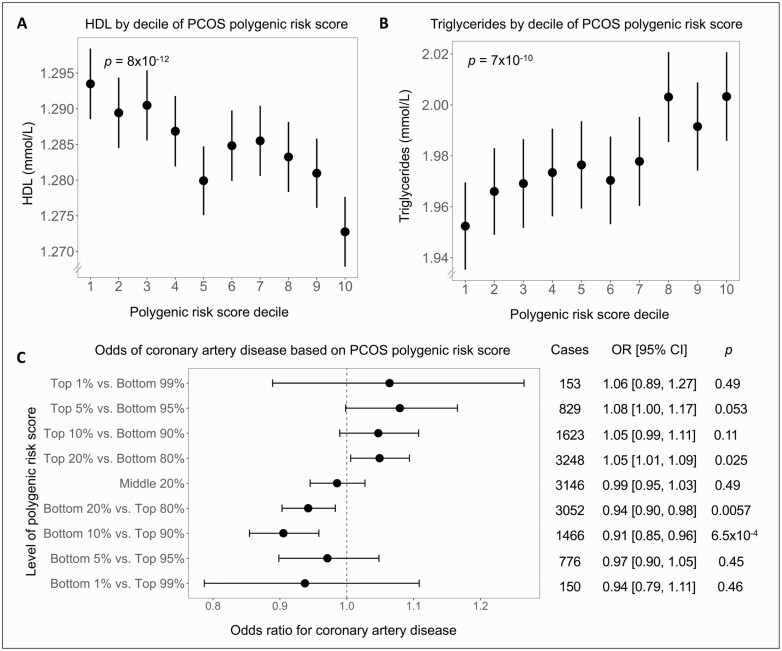
Association of polycystic ovary syndrome (PCOS) polygenic risk score and high-density lipoprotein (HDL), triglycerides, and coronary artery disease. (A) HDL cholesterol decreased and (B) triglycerides increased across deciles of the polygenic risk score for PCOS (*P* values for linear regression shown). (C) Odds ratios of coronary artery disease were calculated by comparing those with a high, middle, or low polygenic risk score with the rest of the cohort using logistic regression. Error bars indicate 95% CI. All analyses were adjusted for age, age squared, genotyping array, and the first 10 genetic principal components.

We next determined the relationship between the PRS and circulating lipids and CAD. A higher PRS was associated with lower high-density lipoprotein (HDL) cholesterol (β = -0.0056 mmol/L or 0.22 mg/dL per 1 SD of PRS, *P *= 8 × 10^-12^) and higher triglycerides (β = 0.018 mmol/L or 1.6 mg/dL per 1 SD of PRS, *P *= 7 × 10^-10^), and the trends were consistent across deciles of PRS (Figs. 3A, 3B). For CAD, we found a 3% increase in odds per 1 SD of PRS (OR: 1.03; 95% CI, 1.01-1.04; *P *= .0029). In addition, those who carried a high PRS had an increased rate of CAD across a range of percentile cutoffs (Fig. 3C).

### Associations Between Polygenic Risk for PCOS and Androgenic Phenotypes in Men

**Figure 4.**
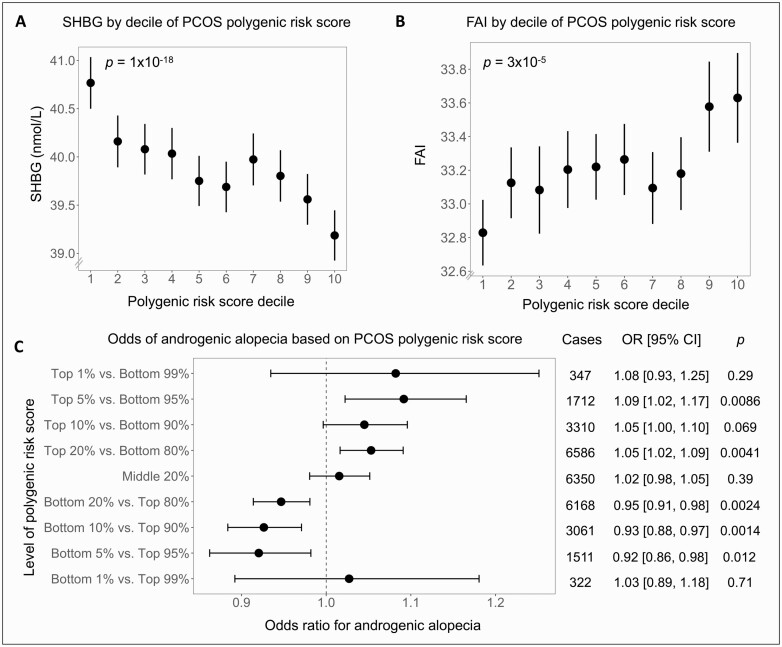
Association of polycystic ovary syndrome (PCOS) polygenic risk score and SHBG, free androgen index (FAI), and androgenic alopecia. (A) SHBG decreased and (B) FAI increased across deciles of the polygenic risk score for PCOS (*P* value for linear regression shown). (C) Odds ratios of androgenic alopecia were calculated by comparing those with a high, middle, or low polygenic risk score with the rest of the cohort using logistic regression. Error bars indicate 95% CI. All analyses were adjusted for age, age squared, genotyping array, and the first 10 genetic principal components.

To assess the effect of polygenic risk for PCOS on androgen levels, we tested the association between PRS and free androgen index (FAI), a measure of androgen bioavailability, and observed that a higher PRS was associated with a higher FAI (β = 0.15 increase in the index per 1 SD of PRS, *P *= 3 × 10^-5^). We also assessed the effect of the PRS on SHBG, which is influenced by androgen action (as well as by obesity) and observed that a higher PRS was associated with a lower SHBG (β = -0.38 nmol/L decrease per 1 SD of PRS, *P *= 1 × 10^-18^). Both the FAI and SHBG trends were consistent across deciles of PRSs (Figs. 4A, 4B).

Next, we assessed the relative prevalence of marked androgenic alopecia (stage 5+ in the Norwood-Hamilton scale) compared with no alopecia (stage 1) in men and observed a 3% increase in odds per 1 SD of PRS (OR: 1.03; 95% CI, 1.02-1.05; *P *= 3 × 10^-5^) and an increased odds of marked androgenic alopecia with higher PRS across a range of percentile cutoffs (Fig. 4C).

### Replication in the EstBB

We sought to replicate the associations between polygenic risk for PCOS and cardiometabolic outcomes in a second, independent dataset of 37 348 unrelated men in the EstBB. In this cohort, we identified 7889 cases and 20 631 controls for obesity, 3595 cases and 10 050 controls for T2DM, and 6161 cases and 31 191 controls for CAD.

Because the sample sizes were smaller than our testing cohort in the UKBB, we had less power to detect associations. Thus, we assessed for consistent trends in the associations between the PRS and the cardiometabolic outcomes and conducted post hoc power analyses to assess our statistical power to detect expected effect sizes.

**Figure 5.**
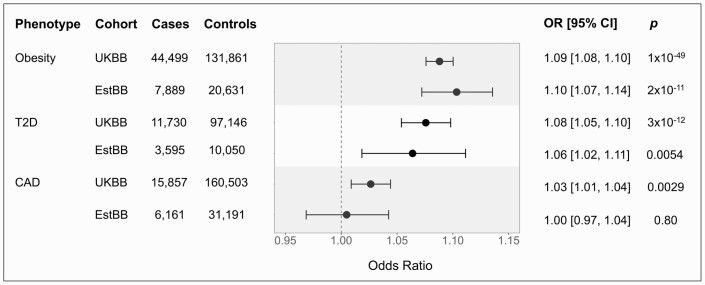
Association of polycystic ovary syndrome (PCOS) polygenic risk score with phenotypes in the UK and Estonian Biobanks. Odds ratios of phenotypes are shown per 1 SD increase in the PCOS polygenic risk score using a logistic regression model. Error bars indicate 95% CI. All analyses were adjusted for age, age squared, genotyping array, and the first 10 genetic principal components. In the UK Biobank, analyses were additionally adjusted for the assessment center; in the Estonian Biobank, analyses were adjusted for batch effects.

Analysis of EstBB replicated the associations between a higher PRS and increased odds of obesity and T2DM that we identified in the UKBB. We observed a 10% increase in odds of obesity (OR: 1.10; 95% CI, 1.07-1.14; *P *= 2 × 10^-11^) and a 6% increase in odds of T2D (OR: 1.06; 95% CI, 1.02-1.11; *P *= .0054) per 1 SD of PRS, which are comparable to the effect sizes in the UKBB (Fig. 5).

For CAD, we did not observe any difference in the odds of disease with increases in the PRS (OR: 1.00; 95% CI, 0.97-1.04; *P *= .80; Fig. 5). A post hoc power analysis showed that our sample size of 6161 cases and 31 191 controls had 80% power to detect an OR ≥ 1.04 per 1 SD of PRS at an α of 0.05. Because the OR that we observed in the UKBB was 1.03, we conclude that our replication analysis of CAD in the EstBB was underpowered to replicate the association observed with the UKBB.

### Mediation by BMI of the Associations Between PCOS Genetic Risk and Cardiometabolic and Androgenic Phenotypes

Increased BMI is a well-known risk factor for PCOS and cardiometabolic diseases, including T2DM and CAD, and has also been linked to androgenic alopecia (3, 22). In our cohort, we observed significant associations between BMI and all evaluated cardiometabolic and androgenic outcomes (*P* all < 1 × 10^-22^ in multiple regression), confirming the significant effect of BMI on these phenotypes. Thus, it is possible that BMI is mediating some or all of the associations between PCOS PRS and cardiometabolic and/or androgenic phenotypes.

We applied mediation analysis to estimate the extent to which BMI mediated the association between the PRS and cardiometabolic or androgenic phenotypes. For example, for HbA1c, there was a significant indirect effect of the PRS on HbA1c attributable to BMI (*P *= 1 × 10^-63^) that accounted for ~66% of the total observed effect. However, even after controlling for BMI, the PRS continued to have a significant direct effect on HbA1c (*P *= .022). Thus, these findings suggest that the relationship between the PRS and HbA1c was partially but not completely mediated by BMI (Table 2). Similarly, the effect of the PRS on T2DM may be partially mediated by BMI (*P *= 2 × 10^-37^ for indirect and *P *= .0011 for direct effect), and the indirect effect attributable to BMI accounted for ~46% of the total observed effect (Table 2).

**Table 2.** BMI as a mediator between PCOS genetic risk score and cardiometabolic and androgenic outcomes

Cardiometabolic phenotypes	Direct effect independent of BMI (95% CI)	P	Indirect effect mediated by BMI (95% CI)	*P*	Estimated proportion of total effect
*Continuous*					
HbA1c (mmol/mol)	0.040 (0.0053-0.074)	.022	0.076 (0.067-0.084)	1 × 10^-63^	0.66
HDL cholesterol (mmol/L)	-0.000834 (-0.0025 to 0.00068)	.27	-0.0047 (-0.0053 to -0.0042)	9 × 10^-65^	0.85
Triglycerides (mmol/L)	0.0039 (-0.0017 to 0.0092)	.18	0.014 (0.012-0.015)	4 × 10^-70^	0.78
*Dichotomous*					
Type 2 diabetes mellitus	0.0033 (0.0013-0.0052)	.0011	0.0028 (0.0024-0.0032)	2 × 10^-37^	0.46
Coronary artery disease	0.00096 (-0.00049 to 0.0023)	.18	0.0010 (0.00089-0.0011)	4 × 10^-58^	0.51
Androgenic phenotypes					
*Continuous*					
SHBG (nmol/L)	-0.15 (-0.23 to -0.073)	.00012	-0.22 (-0.25 to -0.20)	9 × 10^-71^	0.59
Free androgen index	0.095 (0.020-0.17)	.014	0.054 (0.047-0.061)	4 × 10^-50^	0.36
*Dichotomous*					
Androgenic alopecia	0.0061 (0.0026-0.0093)	.00034	0.00076 (0.00059-0.00095)	3 × 10^-16^	0.11

Abbreviations: BMI, body mass index; HbA1c, hemoglobin A1c; HDL, high-density lipoprotein; PCOS, polycystic ovary syndrome.

For HDL cholesterol and triglycerides, there was a significant indirect effect of the PRS on circulating lipids attributed to BMI (*P *= 9 × 10^-65^ for HDL, *P *= 4 × 10^-70^ for triglycerides) that accounted for an estimated 85% and 78% of the total observed effect, respectively. For these outcomes, the PRS no longer had a significant direct effect after controlling for BMI (*P *= .27 for HDL, *P *= .18 for triglycerides), which suggests that the relationships between the PRS and circulating lipids may be completely mediated by BMI. Similarly, mediation analysis suggests that the effect of the PRS on CAD may be completely mediated by BMI (*P *= 4 × 10^-58^ for indirect and *P *= .18 for direct effect; Table 2).

Next, we sought to determine the role of BMI in mediating the relationship between the PRSs and androgenic outcomes. The indirect effect of the PRS on FAI that was attributable to BMI (*P *= 4 × 10^-50^) accounted for ~36% of the total observed effect, and the PRSs continued to have significant direct effects on FAI after controlling for BMI (*P *= .014), which is suggestive of partial mediation by BMI. The effect of the PRS on SHBG may also be partially mediated by BMI (*P *= 9 × 10^-71^ for indirect, *P *= .00012 for direct; Table 2). Because SHBG is influenced by (1) obesity, (2) bioactive androgen levels, and (3) sensitivity to androgens, we sought to isolate the contribution of sensitivity to androgens to the relationship between the PRS and SHBG by controlling for both obesity as measured by BMI and bioactive androgen levels as estimated by FAI. We found a persistent effect of the PRS on SHBG (β = -0.10 nmol/L decrease per 1 SD of PRS; *P *= .0038); this residual effect may be attributable at least in part to sensitivity to androgens.

The effect of the PRS on marked androgenic alopecia may also be partially mediated by BMI (*P *= 3 × 10^-16^ for indirect, *P *= .00034 for direct; Table 2). Androgenic alopecia has also been associated with bioavailable androgen levels (23). Thus, we sought to determine if FAI mediated the association between the PRS and marked androgenic alopecia. The effect of the PRS on marked androgenic alopecia that was indirectly attributed to FAI (*P *= .0060) accounted for ~4.5% of the total observed effect. After controlling for FAI, the PRS continued to have a significant direct effect on marked androgenic alopecia (*P *= 1 × 10^-4^), which suggests that FAI may only partially mediate this relationship.

## Discussion

We found that polygenic risk for PCOS, a diagnosis restricted to women, is associated with cardiometabolic and androgenic conditions and traits in men. Because men do not have ovaries, our findings show that genetic risk factors for PCOS can act independently of ovarian function. Thus, PCOS may not always be a primary disorder of the ovaries.

The polygenic risk for PCOS could have direct effects in men; alternatively, genetic risk factors for PCOS could indirectly affect men through direct effects on female relatives—for example, mothers with PCOS have been proposed to generate an intrauterine environment that increases the cardiovascular risk of offspring (24). If the effects of polygenic risk for PCOS affect men only indirectly, the effects of PCOS polygenic risk would be predicted to be much smaller in men than in women. However, the cardiometabolic effects of polygenic risk for PCOS in men observed in this study are comparable in magnitude to those previously reported in women (25). Similarly, although we cannot exclude the possibility that the association between polygenic risk for PCOS and cardiometabolic and androgenic outcomes are at least partially mediated by ovarian function, the comparable effect sizes between men and women for these outcomes suggests that association between PCOS and these outcomes is largely not mediated by ovarian function.

**Figure 6.**
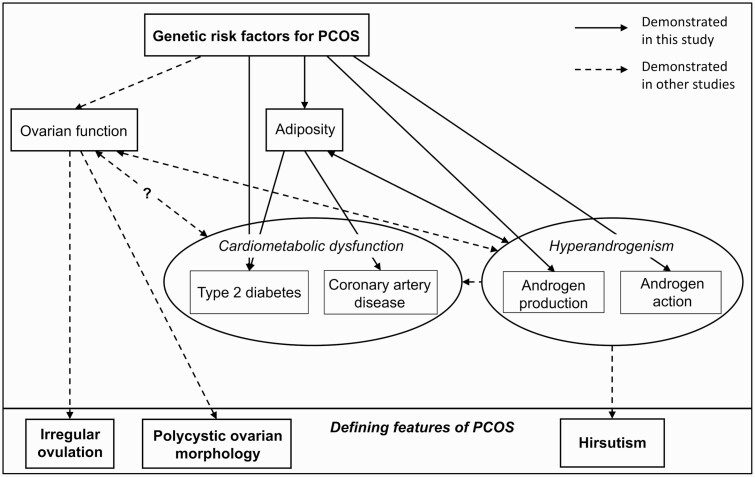
Dissections of the effects of polycystic ovary syndrome (PCOS) genetic risk factors on the phenotypes of PCOS. Illustration of the biological dissections of the PCOS genetic risk factors based on associations with phenotypes in men, including ovary-dependent vs ovary-independent, BMI (adiposity)-dependent vs BMI-independent, and androgen production vs androgen action pathways. Solid arrows depict associations demonstrated in the current study; dotted arrows depict pathways of pathogenesis demonstrated in prior clinical and/or experimental studies.

Moreover, in a prior phenome-wide association study of electronic health records, significant associations between a PRS for PCOS and obesity, morbid obesity, and T2DM in women and men had comparable effect sizes (OR: 1.005-1.010; (25)). Our findings provide further support that the cardiometabolic effects of polygenic risk for PCOS are comparable between men and women and show that the pathogenesis of PCOS may be independent of ovarian function, at least in some cases (Fig. 6).

Furthermore, we show that BMI at least partially mediates the relationship between polygenic risk for PCOS and all cardiometabolic and androgenic phenotypes examined. Specifically, mediation analyses of the effects of polygenic risk for PCOS on HDL cholesterol, triglycerides, and CAD were suggestive of complete mediation by BMI, implying that BMI (or factors reflected by BMI) may be the primary driver in the pathogenesis of dyslipidemia and CAD associated with PCOS. In contrast, the effects of PCOS genetic risk on T2DM and androgenic alopecia suggested a partial mediation by BMI. There is ample evidence that obesity contributes to PCOS, and resolution of obesity can lead to resolution of PCOS in some cases (1). Yet, there are women with PCOS who are not obese. These clinical observations suggest that both obesity-mediated and non-obesity-mediated pathways contribute to PCOS, and the partial mediation by BMI observed in this analysis is consistent with the existence of these 2 types of pathways (Fig. 6, (26)).

Our analysis of androgenic phenotypes provides insights into the biological pathways underlying the pathogenesis of PCOS by implicating both bioactive androgen levels and end-organ responsiveness to androgens as contributors. FAI, an estimate of bioactive androgen levels, only partially mediated the effect of the PRS on androgenic alopecia, which suggests that bioactive androgen levels are not the sole determinant of androgenic alopecia. In agreement, the persistent association between a higher PRS and lower SHBG levels even after controlling for BMI and FAI (factors known to affect SHBG) suggests the additional influence of sensitivity to androgens. Thus, both bioactive androgen levels (systemic and possibly local because of tissue-specific enzyme activity (27)) and the end-organ sensitivity to androgens appear to play a role in the hyperandrogenism of PCOS in both women and men (Fig. 6).

Our PRS for PCOS was associated with both cardiometabolic dysfunction and hyperandrogenism, as indicated by higher levels of FAI. We note that a prior study of individuals in the UKBB reported that genetically determined lower testosterone levels were associated with increased odds of T2DM in men (28). Importantly, the genetic variants in the PCOS PRS were weighted by their strength of association with PCOS in women, in contrast with the variants used in this prior study, which were those most strongly associated with increased testosterone in men. Although the PCOS-associated variants, as we have shown, are also associated with increased FAI, the effect sizes for these variants are different for PCOS than for androgen levels in men. We hypothesize that the PCOS variants that affect free androgen levels have either more complex or more pleiotropic effects, perhaps acting through different mechanisms than the variants selected primarily on the basis of effects on androgen levels in men.

Limitations of our study include the limited predictive power of our PRS, which is constrained by the relatively low number of identifiable cases of PCOS in the UKBB (3). The limited ascertainment of the PCOS phenotype (prevalence of 1.3% compared with population estimates of 6%-10%) in the UKBB may be due to the older age and largely postmenopausal status of the cohort at recruitment (range, 40-69 years) as well as underdiagnosis/underreporting of PCOS. In addition, we were not able to use standards, such as the Rotterdam criteria, that include ovarian morphology to diagnose PCOS because ultrasound results were not available. Thus, it is likely that a portion of women with a history of PCOS were classified as controls in the optimization of our PRS. Despite these limitations, we were able to demonstrate statistically significant effects of polygenic risk for PCOS on clinical outcomes in men, which provide direct genetic evidence that genetic risk factors for PCOS have phenotypic consequences in the absence of ovarian function. Notably, the limited predictive power of our PRS because of the lower-than-expected number of PCOS cases in our cohort may have misestimated effect sizes in our reported outcomes; thus, the true effect sizes may be greater or less than reported. Furthermore, the effect sizes of our cardiometabolic and androgenic outcomes are modest, which may also be due to the limited predictive power of our PRS for PCOS.

Our study provides direct genetic evidence of a correlate to PCOS in men, but the biological mechanisms underlying PCOS in women and its correlate in men remain largely unknown. Identifying the specific genetic variants that are associated with PCOS-related traits will allow categorization of these variants into groups (clusters) by their associated phenotypes. By association testing with specific PCOS phenotypes, genetic data allow for identification of mechanistic pathways driven by different groups of PCOS genetic loci. Classification of these variants and their associated clusters as those that affect both men and women vs those that affect women only will further inform the role of ovarian factors in the pathophysiology of PCOS. These future analyses will allow further refinement of the genetics and molecular pathways that contribute to the pathogenesis of PCOS and bring us closer to identifying future treatment targets of the cardiometabolic dysfunction associated with PCOS for both men and women.

Our results suggest that genetic risk factors for PCOS affect pathological mechanisms of metabolic dysfunction that are common to men and women and that lead to reproductive dysfunction in women. Clinical assessment for genetic risk for PCOS (ie, family history) may inform preventive care for cardiometabolic disease in both sexes. Our findings provide a starting point for dissecting the specific biological pathways underlying the pathogenesis of PCOS, which might aid in the identification of mechanistic pathways, inform clinical subclassification, and lead to future therapeutic targets for PCOS.

## Data Availability

Restrictions apply to the availability of all data generated during this study, which are available to researchers following registration and approval of a research application with the UK and/or Estonian Biobanks.

## Abbreviations

BMI: body mass index
CAD: coronary artery disease
EstBB: Estonian Biobank
FAI: free androgen index
GWAS: genome-wide association study
HbA1c: hemoglobin A1c
OR: odds ratio
PCOS: polycystic ovary syndrome
PRS: polygenic risk score
T2DM: type 2 diabetes mellitus
UKBB: UK Biobank
WHR: waist-to-hip ratio

## Additional Information

***Disclosures*:** The authors disclose no conflicts of interest.
